# Supplementary material for: Urinary proteomics reveals biological processes related to acute kidney injury in Bothrops atrox envenomings
Source: PLoS Negl Trop Dis. 2024 Mar 27;18(3):e0012072. doi: 10.1371/journal.pntd.0012072 (PMC11020875; doi:10.1371/journal.pntd.0012072)
Supplement: Attachment — Submitted filename: Letter urine proteome_210224.docx [file pntd.0012072.s003.docx]

Dear respected reviewers,

We extend our sincere gratitude for dedicating valuable time to review our manuscript and for providing insightful suggestions. We have diligently addressed all the comments raised, striving to enhance the quality of our work for potential publication in PLoS NTD. Enclosed herewith is a comprehensive point-by-point response, detailing the revisions made in accordance with each feedback.

**#Reviewer1**

**1. The method of creatinine and other assays have to be described under the methods.**

Response: Thanks for the reviewer observation. We have increased the Methods section, specifically within item 2.1.2 on Sample Collection and Laboratory Assays. Additional comprehensive details have been incorporated, elaborating on the laboratory assays performed for creatinine, fibrinogen, and D-dimer levels, as follows:

Line 165-178

*“2.1.2 Sample collection and laboratory assays*

*In total, we collected 15 ml of peripheral blood and 60 ml of urine samples from patients admitted to the hospital for snakebite incidents prior to the administration of antivenom. Concurrent with the routine hospital laboratory analyses, we conducted specific analyses to measure the levels of creatinine, fibrinogen, and d-dimer. The blood samples for fibrinogen and d-dimer analyses were collected into Falcon tubes containing 3.8% sodium citrate and 2% pentavalent antivenom, adhering to our internal protocol aimed at neutralizing the venom. The determination of serum creatinine required that patients undergo two measurements: the first prior to antivenom administration and the second at least 48 hours thereafter. For these measurements, we utilized the AA Kinetic Creatinine-liquid assay (Wiener Laboratory, Santa Fe, Argentina). Using an ACL TOP 300 CTS coagulation analyzer (Werfen Instrumentation Laboratory, Barcelona, Spain), we measured fibrinogen levels by the Clauss method [90] and d-dimer levels by immunoturbidimetry. Participants in the control group provided 15 ml of urine and 5 ml of blood, which were used for serum creatinine analysis following the same procedure as for the envenoming cases.”*

**2. According to AKIN criteria, how many of the patients were in stages 1, 2, and 3. That is very important to interpret the proteomics in AKI highly recommended to present the proteomic data according to AKI stages.**

Response: We appreciate the reviewer's insightful observation. To address this concern, we want to clarify that all patients included in our analysis were intentionally diagnosed with Acute Kidney Injury (AKI) at Stage II. This deliberate choice of focusing on a specific stage was made strategically to mitigate potential variability or distortions in the dataset that could arise from including multiple stages of the condition. The decision to concentrate on Stage II was informed by its prevalence within the cohort of snakebite patients monitored during the sample collection period. In response to the reviewer's suggestion, we have enriched the Methods section of our manuscript with detailed information about the AKI stage. This clarification has been explicitly stated in the captions of both Table 1 and Table 2, emphasizing that our study observations were confined to a single stage of AKI. Furthermore, we have acknowledged the limitation of examining only one stage of AKI in our study, ensuring a transparent and comprehensive understanding of our research approach and its potential implications (see below).

Lines 154 -161

*“AKI diagnosis and staging followed the Acute Kidney Injury Network (AKIN) criteria [21]. The patients who had a primary change in serum creatinine of ≥0.3mg/dL or ≥150 to 199% on their baseline value were considered to have AKI clinical outcomes. The stages of kidney injury according to AKIN [21] consider Stage 1 lesion - an increase in serum creatinine of > 0.3mg/dL or ≥ 150 to 199% about baseline creatinine, Stage 2 lesion - an increase in 200 to 299%, and Stage 3 Lesion – an increase of ≥ 4.0mg/dL or ≥ 300% or patient starting Renal Replacement Therapy. To minimize data variability, only patients classified with AKI at Stage 2 lesion were selected for proteomic analysis.”*

Lines 569-573

*“Thus, the results of this work are also limited by the small size cohort of AKI patients, the few female patients observed, the presence of AKI patients with hypertension, and the analysis of only AKI at Stage 2; thus, further studies with larger cohorts observing these aspects are needed to validate and reinforce the results presented in our study.”*

**3. What is the sample collection timing of AKI and non-AKI groups following snakebite? These data should be well presented under the results. If sample collection happened sooner after the bite in the non-AKI group, some of these patients may develop AKI alone the time course. This would significantly affect the differentiation of two groups.**

Response: We sincerely appreciate the insightful consideration provided by the reviewer regarding the timing of sample collection for the AKI and non-AKI groups following snakebite. We want to unequivocally emphasize the meticulous approach we adopted to synchronize sample collection precisely with the admission of patients to the hospital – a critical window for data acquisition. It is imperative to underscore that, for nearly 90% of individuals in both cohorts, the duration from the snakebite incident to hospital admission did not exceed **six hours**. This crucial detail is thoughtfully delineated in Table 1, titled "Demographic and Clinical Characteristics of Bothrops Snakebite Patients at Admission," specifically under the 'Time to Admission' section. Indeed, the deliberate alignment in the timing of sample collection is not just a procedural detail; it is a strategic strength of our study. By eradicating the possibility of disparate collection intervals, we have significantly bolstered the internal validity of our findings. Furthermore, it is noteworthy that all participants received immediate and uniform treatment at the same healthcare facility, underscoring the consistency and timeliness of medical intervention. We trust that these details compellingly support the rigor of our study design and the credibility of our conclusions.

**4. Describe the other clinical features between AKI and non-AKI groups and present under the results.**

Response: In alignment with the provided suggestion, we have seamlessly incorporated Supplementary Material File 1 into the main article, presenting its contents as Table 1 and Table 2. These tables comprehensively outline the demographic and clinical characteristics of Bothrops snakebite patients at admission and provide detailed information on the biochemical laboratory results, respectively, as bellow:

*A total of 30 patients were included in the analysis, equally distributed among the three groups evaluated: healthy controls, Bothrops snakebite patients who developed acute kidney injury (AKI), and those who did not (No-AKI). A comprehensive summary of the demographic, clinical, and laboratory characteristics of the cohort can be found in Tables 1 and 2.*

Table 1. Demographic and clinical characteristics of Bothrops snakebite patients at admission.

| **Variable** | Total (n=20) | **No-AKI** (n=10) | **AKI*** (n=10) |
| --- | --- | --- | --- |
| **Age range (years)** |  |  |  |
| 16-45 | 13 (65.0%) | 8 (80.0%) | 5 (50.0%) |
| 45-60 | 3 (15.0%) | 1 (10.0%) | 2 (20.0%) |
| >60 | 4 (20.0%) | 1 (10.0%) | 3 (30.0%) |
| **Sex** |  |  |  |
| Male | 17 (85.0%) | 9 (90.0%) | 8 (80.0%) |
| **Occurrence zone** |  |  |  |
| Rural | 19 (95.0%) | 10 (100.0%) | 9 (90.0%) |
| Urban | 1 (5.0%) | 0 (0.0%) | 1 (10.0%) |
| **Time to medical assistance** |  |  |  |
| <6 hours | 18/20 (90.0%) | 9/10 (90.0%) | 9/10 (90.0%) |
| **Site of bite** |  |  |  |
| Foot | 12/20 (60.0%) | 6/10 (60.0%) | 6/10 (60.0%) |
| Leg | 6/20 (30.0%) | 3/10 (30.0%) | 3/10 (30.0%) |
| Hand | 2/20 (10.0%) | 1/10 (10.0%) | 1/10 (10.0%) |
| **Previous snakebite** | 4/20 (20.0%) | 0/10 (0.0%) | 4/10 (40.0%) |
| **Use of tourniquet** | 2/20 (10.0%) | 1/10 (10.0%) | 1/10 (10.0%) |
| **Use of traditional medicines** | 5/20 (25.0%) | 1/10 (10.0%) | 4/10 (40.0%) |
| **Severity classification** |  |  |  |
| Mild | 3/20 (15.0%) | 2/10 (20.0%) | 1/10 (10.0%) |
| Moderate | 11/20 (55.0%) | 4/10 (40.0%) | 7/10 (70.0%) |
| Severe | 6/20 (30.0%) | 4/10 (40.0%) | 2/10 (20.0%) |
| **Comorbidities** | 4/20 (20.0%) | 0/10 (0.0%) | 4/10 (40.0%) |
| Chronic gastric | 1/4 (25.0%) | 0/10 (0.0%) | 1/4 (25.0%) |
| Hypertension | 3/4 (75.0%) | 0/10 (0.0%) | 3/4 (75.0%) |
| **Concomitant medication** |  |  |  |
| Amlodipine | 1/4 (25.0%) | NA | 1/4 (25.0%) |
| Losartan | 2/4 (50.0%) | NA | 2/4 (50.0%) |
| Omeprazole | 1/4 (25.0%) | NA | 1/4 (25.0%) |
| **Local manifestations** | 8/20 (40.0%) | 5/10 (50.0%) | 3/10 (30.0%) |
| Bleeding | 6/20 (30.0%) | 4/10 (40.0%) | 2/10 (20.0%) |
| Edema | 6/20 (30.0%) | 4/10 (40.0%) | 2/10 (20.0%) |
| Ecchymosis | 1/20 (5.0%) | 1/10 (10.0%) | 0/10 (0.0%) |
| Blisters | 2/20 (10.0%) | 0/10 (0.0%) | 2/10 (20.0%) |
| Necrosis | 1/20 (5.0%) | 1/10 (10.0%) | 0/10 (0.0%) |
| **Systemic manifestations** | 9/20 (45.0%) | 5/10 (50.0%) | 4/10 (40.0%) |
| Headache | 5/20 (25.0%) | 3/10 (30.0%) | 2/10 (20.0%) |
| Diarrhea | 1/20 (5.0%) | 0/10 (0.0%) | 1/10 (10.0%) |
| Vomit | 1/20 (5.0%) | 1/10 (10.0%) | 0/10 (0.0%) |
| Nausea | 2/20 (10.0%) | 0/10 (0.0%) | 2/10 (20.0%) |
| Gum bleeding | 2/20 (10.0%) | 2/10 (20.0%) | 0/10 (0.0%) |

NA – Not Applicable. *All AKI patients were diagnosed at Stage 2 Lesion following AKIN criteria.

**Table 2.** Laboratory characterization of the patients at admission.

| **Variable** | Total (n=20) | **No-AKI (n=10)** | **AKI (n=10)** |
| --- | --- | --- | --- |
| **Creatinine** |  |  |  |
| Normal | 12/20 (60.0%) | 10/10 (100.0%) | 2/10 (20.0%) |
| High value | 8/20 (40.0%) | 0/10 (0.0%) | 8/10 (80.0%) |
| **Creatinine mean (SD)** | 1.2 (0.6) | 0.8 (0.2) | 1.5 (0.7) |
| **Urea** |  |  |  |
| Normal | 16/20 (80.0%) | 10/10 (100.0%) | 6/10 (60.0%) |
| High value | 4/20 (20.0%) | 0/10 (0.0%) | 4/10 (40.0%) |
| **Urea mean (SD)** | 38.2 (19.1) | 28.8 (8.2) | 47.5 (22.6) |
| **Lactate dehydrogenase** |  |  |  |
| Normal | 8/15 (53.3%) | 4/6 (66.7%) | 4/9 (44.4%) |
| High value | 7/15 (46.7%) | 2/6 (33.3%) | 5/9 (55.6%) |
| **Lactate dehydrogenase mean (SD)** | 600.4 (590.9) | 440.3 (152.7) | 707.1 (751.3) |
| **Creatine phosphokinase** |  |  |  |
| Normal | 9/14 (64.3%) | 3/4 (75.0%) | 6/10 (60.0%) |
| High value | 5/14 (35.7%) | 1/4 (25.0%) | 4/10 (40.0%) |
| **Creatine phosphokinase mean (SD)** | 218.3 (199.4) | 134.8 (77.2) | 251.7 (226.0) |
| **Clotting time** |  |  |  |
| Normal | 7/20 (35.0%) | 4/10 (40.0%) | 3/10 (30.0%) |
| Prolonged | 1/20 (5.0%) | 0/10 (0.0%) | 1/10 (10.0%) |
| Unclottable | 12/20 (60.0%) | 6/10 (60.0%) | 6/10 (60.0%) |
| **Prothrombin activity time** |  |  |  |
| Normal | 3/19 (15.8%) | 2/9 (22.2%) | 1/10 (10.0%) |
| Prolonged | 11/19 (57.9%) | 5/9 (55.6%) | 6/10 (60.0%) |
| Unclottable | 5/19 (26.3%) | 2/9 (22.2%) | 3/10 (30.0%) |
| **Hemoglobin** |  |  |  |
| Normal | 14/20 (70.0%) | 7/10 (70.0%) | 7/10 (70.0%) |
| High | 4/20 (20.0%) | 2/10 (20.0%) | 2/10 (20.0%) |
| Low | 2/20 (10.0%) | 1/10 (10.0%) | 1/10 (10.0%) |
| **Hemoglobin mean (SD)** | 14.2 (2.6) | 13.9 (3.3) | 14.5 (1.7) |
| **Leukocytes** |  |  |  |
| Normal | 8/20 (40.0%) | 4/10 (40.0%) | 4/10 (40.0%) |
| High | 12/20 (60.0%) | 6/10 (60.0%) | 6/10 (60.0%) |
| **Leukocytes mean (SD)** | 11483.3 (4643.6) | 11602.3 (5958.0) | 11364.2 (3161.1) |
| **Platelets** |  |  |  |
| Normal | 19/20 (95.0%) | 10/10 (100.0%) | 9/10 (90.0%) |
| Low | 1/20 (5.0%) | 0/10 (0.0%) | 1/10 (10.0%) |
| **Platelets mean (SD)** | 203538.0 (51744.2) | 218880.0 (33080.2) | 188196.0 (63522.1) |
| **Fibrinogen** |  |  |  |
| Normal | 5/20 (25.0%) | 3/10 (30.0%) | 2/10 (20.0%) |
| High | 2/20 (10.0%) | 1/10 (10.0%) | 1/10 (10.0%) |
| Low | 13/20 (65.0%) | 6/10 (60.0%) | 7/10 (70.0%) |
| **D-dimer** |  |  |  |
| Normal | 2/19 (10.5%) | 2/9 (22.2%) | 0/10 (0.0%) |
| High | 17/19 (89.5%) | 7/9 (77.8%) | 10/10 (100.0%) |
| **Urine hemoglobin** | 5/10 (50.0%) | 0/2 (0.0%) | 5/8 (62.5%) |
| **Urine leukocytes** | 3/10 (30.0%) | 1/2 (50.0%) | 2/8 (25.0%) |
| **Urine crystals** | 1/10 (10.0%) | 0/2 (0.0%) | 1/8 (12.5%) |
| **Urine casts** | 6/10 (60.0%) | ½ (50.0%) | 5/8 (62.5%) |

**Reference values**: Lee-White clotting time: ≤ 9 min; Prothrombin activity time: 13 secs; Creatinine: 0.5 ± 1.2 mg/dL; Lactate dehydrogenase: 211 ± 423 IU/L; Creatine phosphokinase: 24 ± 190 IU/L; Leukocytes: 4,000 ± 10,000/mm3; Fibrinogen: 180 ± 350 mg/dL; D-dimer: < 500 ng/dL. Hemoglobin: 12.5±15.5 g/dL; Leukocytes: 4,000±10,000/mm^3^; Platelets: 150,000±450,000/mm^3^; Fibrinogen: 180±350 mg/dL; D-dimer: <500ng/dL. *All AKI patients were diagnosed at Stage 2 Lesion following AKIN criteria.

**5. Line 266: 75% had hypertension: How authors can attribute the changes in proteomics happened to the snakebite, and not due to hypertension? What type of proteomics baseline reported due to hypertension, and this has to be discussed in details in the discussion.**

Response: Firstly, we appreciate the meticulous observation and would like to rectify a miscommunication in our manuscript. The statement "75% of AKI patients had hypertension" was inaccurately phrased. Among the 10 patients in the AKI group, 4 had comorbidities, and 3 out of these 4 patients (75%) had hypertension. To provide a clearer perspective, this comorbidity was present in only 30% of the total AKI patients (10) and 15% of the overall snakebite victims (20) included in our study. This correction has been accurately reflected in Table 1, where we detail the demographic, clinical, and epidemiological information of snakebite patients.

Secondly, recognizing the potential impact of hypertension as a source of bias, we have transparently acknowledged this limitation in our work. We acknowledge the inability to establish a baseline for the effects of hypertension on the proteomic profile of AKI patients due to the constraints on excluding these patients from the AKI envenomed cohort. This limitation is explicitly addressed in the manuscript, emphasizing our commitment to transparency in research reporting.

Finally, to address the concern about attributing urine proteomic changes specifically to snakebite, we want to highlight our study design. We meticulously observed a control group without venom influence, serving as a baseline to understand what can be expected from 'normal' urine under our experimental conditions. This control group's proteins and their abundance were utilized as a benchmark to attribute changes observed in the AKI group to the snakebite itself, despite the presence of patients with hypertension. This information is detailed in the manuscript, emphasizing the robustness of our study design in isolating, and attributing proteomic changes to the snakebite.

See lines 287-292

*“In overview, the population analyzed predominantly comprises men (90%), from rural areas (95%), with hypertension as the main comorbidity found among the envenoming victims (15% of patients). Furthermore, 20% of the individuals had experienced previous snakebites, and 55% suffered moderate accidents. Interestingly, despite 90% of the patients receiving medical treatment within less than 6 hours, 50% of this population developed AKI according to the AKIN criteria.”*

**6. Line 266: What does meant by "moderate accidents"?**

Response: "Moderate accidents" corresponds to a specific classification within the framework defined by the Guidelines of the Brazilian Ministry of Health for categorizing the severity of snakebites. This classification is based on a thorough evaluation considering factors such as the snake species involved, clinical manifestations observed at the time of hospital admission, and the coagulation profile of the patient. In our study, moderate cases are characterized by the presence of pain and significant edema extending across three to four body segments. Furthermore, these cases may exhibit coagulopathy or other systemic manifestations, such as gingival bleeding, without compromising the overall condition of the patient.

To enhance clarity and provide a detailed understanding of the criteria used for accident classification, we have incorporated comprehensive information into the Methods section of our manuscript, specifically under subsection 2.1 Study Design as below:

Lines 144-153

***“****The snakebite clinical severity was classified on hospital admission according to the Brazilian Ministry of Health Guidelines for Snakebite Diagnosis and Treatment [2] as mild cases, characterized by pain and/or local edema, affecting one or two body segments, may present coagulopathy, without distant bleeding; moderate cases, characterized by pain and evident edema affecting three to four body segments, may present coagulopathy or other systemic symptoms, such as gingival bleeding, but without impairment of general condition, and severe cases, characterized by edema involving the entire affected limb (5 body segments), severe pain, may present coagulopathy or other systemic symptoms, such as hypotension, may also present distant bleeding, necrosis, and/or compartment syndrome.”*

**7. Two different citation styles have been used to cite the references in the text. Please correct it according to PLoS citing style.**

Response: Thank you for bringing attention to the inconsistency in citation styles. We have addressed this issue and ensured that the citation styles are now consistent and adhere to the PLoS citing style throughout the manuscript. work.

**8. There are many discussion points have been included and discussed under the results sections and cited the relevant references. Since the article style has a separate section for the discussion, please move all the discussion points included in the results to under the discussion section. State only the results of THIS study under the discussion.**

Response: We have reorganized the manuscript by moving all discussion points that were initially placed under the results section to the appropriate section dedicated to the discussion. This adjustment ensured a more structured and coherent presentation.

**9. Line 161:is it micro or millilitres?**

Response: It is measured in milliliters. We have rectified this oversight and made the necessary correction to ensure accuracy in the unit of measurement throughout the manuscript.

**11. Line 48, 49 and few more places: "Bothrops" should be spelled as "Bothrops"**

Response: Thank you for highlighting the inconsistency in the capitalization of "Bothrops." We have duly made the correction, and the manuscript has been thoroughly revised to ensure uniformity in the spelling.

**Reviewer #2:**

**12. More details are needed to explain the inclusion and exclusion criteria used in this study to recruit the patients.**

Response: Thank you for highlighting that. To address this concern, we have incorporated comprehensive information in the Methods section, specifically under 2.1 Study Design, elaborating on the specific criteria utilized for patient recruitment. This addition aims to provide a thorough understanding of the parameters guiding the inclusion and exclusion of patients in our study. See below:

Lines 134-140

*“... The inclusion criteria for snakebite patients were individuals of any sex or age, victims of Bothrops envenoming, without prior treatment, with at least two measures of serum creatinine, being the first before the antivenom and the second at least 48h after the first one. We excluded patients with diabetes mellitus, renal diseases, autoimmune disease, and continuously using non-steroidal anti-inflammatory drugs.”*

**13. How did the authors ascertain AKI in these patients? Which methods were used? what stages of AKI were developed in these patients in line with AKIN criteria?**

Response: We greatly appreciate the reviewer insightful inquiry into the methods employed to ascertain Acute Kidney Injury (AKI) in our study. In response, we have provided detailed information under the Methods section, including the laboratory techniques used for measuring serum creatinine, the time points of sample collection, and an expanded explanation of the parameters based on the AKIN criteria for different stages of AKI. To minimize variability within the data analyzed, we selected only patients classified with AKI at Stage 2 lesion for proteomic analysis. This intentional selection enhances the homogeneity of the data analyzed, providing a more robust examination of the proteomic changes associated specifically with the condition evaluated. We trust that these additions significantly reinforce the clarity and robustness of our study methodology.

See below for the updated text in the manuscript.

See lines 154-161

*“AKI diagnosis and staging followed the Acute Kidney Injury Network (AKIN) criteria [21]. The patients who had a primary change in serum creatinine of ≥0.3mg/dL or ≥150 to 199% on their baseline value were considered to have AKI clinical outcomes. The stages of kidney injury according to AKIN [21] consider Stage 1 lesion - an increase in serum creatinine of > 0.3mg/dL or ≥ 150 to 199% about baseline creatinine, Stage 2 lesion - an increase in 200 to 299%, and Stage 3 Lesion – an increase of ≥ 4.0mg/dL or ≥ 300% or patient starting Renal Replacement Therapy. To minimize data variability, only patients classified with AKI at Stage 2 lesion were selected for proteomic analysis.”*

**14. What is the likelihood of patients having undiagnosed previous health conditions including AKI? It would be helpful to address all these points with further details.**

Response: We appreciate the reviewer's valid concern about the possibility of patients having undiagnosed previous health conditions, including Acute Kidney Injury (AKI). While it is acknowledged that individuals at higher risk of snakebites may also be susceptible to other neglected tropical diseases that could go undiagnosed, we want to emphasize the robustness of our approach in mitigating this potential bias.

We conducted thorough data collection through patient interviews, examination of previous medical records, and laboratory assays during admission, aiming to capture a comprehensive overview of the patients' health status. Importantly, for a patient to have undiagnosed previous AKI, they would likely exhibit symptoms associated with kidney dysfunction, such as changes in urination patterns, fluid retention, or fatigue. Given the acute and often symptomatic nature of AKI, the likelihood of it being undiagnosed without noticeable symptoms is considerably low. Moreover, AKI is characterized by a sudden onset of kidney failure or damage, typically occurring within a few hours or days. It is a condition that often presents with evident symptoms and is reversible with prompt treatment. Furthermore, if the impairment persists beyond three months, it is considered a chronic kidney disease, falling under the 'renal disease' exclusion criteria of our study.

By underscoring the symptomatic nature of AKI and detailing our thorough data collection methods, we believe our study design effectively addresses concerns about undiagnosed previous health conditions, enhancing the credibility and reliability of our findings.

**15. At what time points, serum creatinine was measured? when was the second one? At what stage does the creatinine level get elevated to ascertain AKI in bothrops envenomings?**

Response: The diagnosis of acute kidney injury (AKI) in our study is established when there is an observed change in serum creatinine (sCrea) of ≥0.3mg/dL or ≥150 to 199% compared to their baseline value. The baseline value is determined with the initial measurement of sCrea conducted at the time of hospital admission, and the second measurement occurs 48 hours after the first dosage.

In response to both reviewers' suggestions, we have expanded the information regarding the criteria and laboratory methods used for AKI diagnosis. It is crucial to note that the criteria and protocol employed in our study are not specific to snakebite envenoming conditions. Instead, they are in accordance with guidelines established by the Acute Kidney Injury Network (AKIN), widely utilized in hospital routine. AKIN is an independent collaborative network of experts from various societies dedicated to clinical and basic research in kidney physiology and diseases, including acute renal failure. Notable associations include the Acute Dialysis Quality Initiative (ADQI) group, the American Society of Nephrology (ASN) ARF Advisory group, the International Society of Nephrology (ISN), the National Kidney Foundation (NKF), and KDIGO (kidney disease: Improving Global Outcomes). It is important to clarify that our study's primary objective was to describe the urinary proteomic profile of AKI within the context of snakebite envenoming. While the question of how well the AKIN criteria perform in diagnosing AKI in this specific scenario is interesting, we believe it goes beyond the intended scope of our work. Nevertheless, we appreciate the valuable input and have taken steps to provide a more comprehensive understanding of our AKI diagnosis methodology within the constraints of our study objectives.

See lines 134-138:

*“The control group was conveniently sampled from healthy individuals with sex and age, pairing the snakebite victims' profiles. The inclusion criteria for snakebite patients were individuals of any sex or age, victims of Bothrops envenoming, without prior treatment, with at least two measures of serum creatinine, being the first before the antivenom and the second at least 48h after the first one.*

See lines 154-161:

*“AKI diagnosis and staging followed the Acute Kidney Injury Network (AKIN) criteria [21]. The patients who had a primary change in serum creatinine of ≥0.3mg/dL or ≥150 to 199% on their baseline value were considered to have AKI clinical outcomes. The stages of kidney injury according to AKIN [21] consider Stage 1 lesion - an increase in serum creatinine of > 0.3mg/dL or ≥ 150 to 199% about baseline creatinine, Stage 2 lesion - an increase in 200 to 299%, and Stage 3 Lesion – an increase of ≥ 4.0mg/dL or ≥ 300% or patient starting Renal Replacement Therapy. To minimize data variability, only patients classified with AKI at Stage 2 lesion were selected for proteomic analysis.”*

**16. In some places, the snake genus or species names are not in italics.**

Response: Thank you for bringing this to our attention. We have thoroughly reviewed the entire manuscript and ensured that all snake genus and species names are appropriately formatted in italics for consistency and adherence to standard formatting conventions.

**17. I would suggest the authors give more focus on the actual mass spec data obtained in this study. For example, which parameters were used to differentiate the proteins from baseline noise to full proteins/peptides? was the abundance of each protein clearly quantified? It's better to explain these details in more detail compared to the predicted results in the later sections.**

17.1 **I would suggest the authors give more focus on the actual mass spec data obtained in this study. For example, which parameters were used to differentiate the proteins from baseline noise to full proteins/peptides?**

Response: Thank you for highlighting the need for more information on the actual mass spectrometry data obtained in this study. To provide a comprehensive understanding, it’s important to introduce some concepts before we explain what parameters were used to differentiate the proteins from baseline noise to full proteins/peptides. To interpret the mass spec data and convert it to peptides/protein identification we applied a peptide spectrum match (PSM) search approach using the Comet software. In this search strategy, we build a database encompassing the peptide sequences from the consensus proteome of all organisms evaluated in the study, sequences of 123 most common contaminants that might appear in a mass spectrometry proteomic analysis, and a decoy sequence database created by reversing the target database sequences. This decoy database aids in controlling the false discovery rate (FDR) by introducing biologically nonexistent sequences that share statistical attributes with the target sequences. In general terms, Comet generates theoretical mass spectra based on given sequences and matches them against real spectra from the experiment. To perform this matching, Comet follows a set of multiple technical parameters to differentiate the peptide sequences from the baseline noise. These parameters consider factors such as sample preparation settings (e.g., enzyme specificity, missed cleavages), amino acid modifications, and mass spectrometer configurations (e.g., fragment bin tolerance, mass range search). These parameters guide the software in ‘how to read’ the experimental spectra obtained and how to match them with the theoretical spectra from the target-decoy database. In our work, the resulting list of identifications undergoes further refinement through a filtering process using the Search Engine Processor (SEPro) from Pattern Lab for Proteomics V software. SEPro employs a three-tier Bayesian approach that uses layers of spectrum, peptide, and protein logic to lead the data to converge to a single list of reliable protein identifications.

It filters considering parameters of protein scores, a minimum sequence length of residues, and mass deviations from the theoretical mass. Then, the list of identification is submitted to the application tools for quantitation and data analysis, all of them containing their own setting of parameters. The settings for these parameters were outlined succinctly in the first version of our article under Methods, with false discovery rates provided under Results. To enhance clarity about this process, we have updated the Methods section, describing the settings and logic behind each step of the mass spectrometry data processing.

Finally, we recognize the importance of detailing the technical aspects, however the explanation for the chosen settings of each one of these parameters can be lengthy and technical heavy. Since our focus in this work was to explore AKI on snakebite envenoming by applying well-established proteomics methods and tools, we believe adding further details of these over discussing the biological interpretation of the results found may mischaracterize the scope of the current work. Finally, we hope these revisions offer a more comprehensive overview of our mass spectrometry data processing methodology.

See below changes in the manuscript 227 - 279

*“2.2.4 Search and identification of spectral data*

*The data analysis for protein identification and relative quantitation was performed using the software PatternLab for Proteomics V (PLV), freely available at http://www.patternlabforproteomics.org [28]. For peptide/protein sequence identification, we applied a peptide spectrum match approach (PSM) using the search engine Comet 2021.01 rev. 0 [29] for identification of peptide/protein from the mass spectra data. The target-decoy database was prepared by including peptide sequences from Homo sapiens and Bothrops atrox, plus sequences from the 123 most common mass spectrometry contaminants for protein analysis, and a decoy database generated through reversion of each sequence from the target database [28]. The sequences from H. sapiens were downloaded on March 23rd, 2023, from the Swiss-Prot database. The B. atrox sequences were obtained from a transcriptome database kindly provided by collaborators at Butantan Institute. The search parameters for identification were configured at the High-High option [29], considering fully and semi-tryptic peptide candidates with mass ranging from 500 to 6,000 Da, up to two missed cleavages, 35 ppm for precursor mass, and fragment ion bins of 0.02 m/z for MS/MS. The amino acid residue modifications evaluated were carbamidomethylation of cysteine as fixed, oxidation of methionine, and carbamylation of lysine and arginine as variable.*

*2.2.5 Validation of PSMs*

*The Search Engine Processor (SEPro) was used to assess the validity of PSMs [30]. The identifications were grouped by charge state (2+ and ≥ 3+), and then by tryptic status, generating four distinct subgroups. The XCorr, DeltaCN, DeltaPPM, and Peaks Matches values were used to produce a Bayesian discriminator, in each group. The identifications were sorted in nondecreasing order according to the discriminator score. A false-discovery rate (FDR) of 2% at the peptide level based on the number of decoys was accepted for the cutoff score. This process was independently executed on each data subset, resulting in an FDR independent of charge state or tryptic status. Additionally, as filtering parameters, we required a protein score greater than three, and a minimum sequence length of six amino-acid residues. Lastly, identifications deviating by more than 10 ppm from the theoretical mass were discarded.”*

17.2 **Was the abundance of each protein clearly quantified**?

Response: The quantitation of protein abundance in proteomics studies can be approached through relative or absolute quantitation, with the latter mainly involving labeling methods. Labeling methods are considered the gold standard for their superior accuracy and reproducibility compared to label-free methods. Among the label-free quantitation protocols, the extracted ion chromatogram (XIC) strategy provides the higher level of accuracy. In our study, we employed a label-free protocol utilizing the XIC approach, and we acknowledge the inherent limitations in the accuracy implied by the method used. Despite these limitations, we were able to clearly quantify the abundance of 1,190 proteins that fitted the criteria for two unique peptides. Additionally, label-free approaches offer distinct advantages over labeling methods, including the identification of a larger number of proteins across a broader dynamic range, ease of result comparison with a greater number of studies due to the prevalence of label-free experiments, and a simpler, faster, and less expensive workflow. Given our infrastructure capabilities and the specific objectives of our study, we deemed the use of a label-free protocol sufficient to achieve the desired exploration of the protein profile in snakebite victims who developed AKI. We have incorporated comments on the quantitation approach in the limitations section, as suggested, to provide further context and transparency regarding the methodology's constraints.

See lines 587- 599

*“Regarding limitations on protein identification, we were not able to identify Bothrops venom-derived protein or peptide in the urine samples. This lack of identification derives from the challenges of studying a non-model organism due to fewer curated gene or transcript sequence databases [26,32,33]. In addition to this, the diversity of toxins proteoforms in snake venoms presents an additional challenge to bottom-up analysis of complex samples, such as those analyzed in this study [26,34]. Lastly, it’s noteworthy that absolute quantitation through labeling methods are considered the gold standard for quantitation in proteomics due to their higher accuracy and reproducibility when compared to label-free methods [94]. We performed a label-free protocol with relative quantitation by the extracted ion chromatogram (XIC) strategy. This means our quantitative results, and the conclusions derived from them, are impacted by the limitations on the accuracy of this method's use. Still, among the label-free quantitation protocols, the approach we applied - extracted ion chromatogram (XIC)- has the greater accuracy [94]. In addition, the use of a label-free protocol presents the benefit of identifying a larger number of proteins with a wider dynamic range of detection, when compared to labeling-methods [95]. We believe the use of a label-free protocol was sufficient to achieve the intended aim of exploring the protein profile of snakebite victims who developed AKI, an still understudied topic. “*

**18. Would it be possible to correlate the mass spec data with specific parameters of patients? for example, male patients Vs females? different age categories? time of bite? or time to antivenom? or any of their clinical symptoms or hematological parameters? This correlation will be helpful to link these protein compositions to the actual manifestations.**

Response: Given the small cohort of patients and the study's design, establishing a direct quantitative correlation between proteomic data and patients' clinical parameters beyond the intended focus on acute renal lesions is challenging. Previous research has noted variations in urine composition related to gender and age, and snakebite studies emphasize the impact of the time between the bite and antivenom treatment on complications such as AKI. To mitigate potential variables affecting the analysis of the primary subject – AKI in snakebite victims – we took several measures. These included pairing age and gender between the evaluated groups, restricting the intervals between accidents and hospital admission, and specifically examining one AKI stage. To elucidate these considerations in the article, we have expanded the discussion of the limitations in this work and provided additional details under the Study Design section in the Methods. Recognizing the importance of the questions raised by Reviewer #2, our ongoing research efforts now focus on individually evaluating the molecular aspects related to some of these parameters. We appreciate the insightful feedback and have taken steps to transparently address the limitations and complexities inherent in our study design.

See lines 134 – 143 in the Study design and lines 563-580 in the discussion section.

***“2.1 Study design***

*For descriptive comparison matters, three groups of samples were submitted to proteomic analysis: healthy controls (n=10), Bothrops snakebite victims that developed acute kidney injury (AKI, n=10), and patients that did not develop this condition (No-AKI, n=10).*

*The control group was conveniently sampled from healthy individuals with sex and age, pairing the snakebite victims' profiles. The inclusion criteria for snakebite patients were individuals of any sex or age, victims of Bothrops envenoming, without prior treatment, with at least two measures of serum creatinine, being the first before the antivenom and the second at least 48h after the first one. We excluded patients with diabetes mellitus, renal diseases, autoimmune disease, and continuously using non-steroidal anti-inflammatory drugs. We considered the accidents as caused by Bothrops through recognition of clinical symptoms of Bothrops envenoming or snake identification by a trained herpetologist when possible. As an additional confirming step, we performed a venom antigenemia assay consisting of an ELISA with Mab anti-B. atrox plus biotin.”*

“**4. Discussion**

*Moreover, the naturally high variability in urine poses a challenge for analyzing these samples, particularly for biomarker research [19,20,51]. Various factors are already known to influence the urinary protein landscape, such as the patient’s sex and age, the timing of sample collection, the presence of comorbidities, dietary composition, the volume of venom inoculated, and even the time between the bite and hospital care [22,68,89]. Thus, the results of this work are also limited by the small size cohort of AKI patients, the few female patients observed, the presence of AKI patients with hypertension, and the analysis of only AKI at Stage 2; thus further studies with larger cohorts observing these aspects are needed to validate and reinforce the results presented in our study. These limitations cited impose still unknown effects on the data presented, especially the presence of hypertensive patients. Hypertension frequently causes renal damage, the extension of it it’s correlated to several factors, such as the individual susceptibility, the degree of hypertension, or even if it's being properly treated or not [91]. These possible injuries might lead to a higher abundance of some proteins and create a bias for these in the data. However, hypertension is a common comorbidity in the general population and among AKI patients [93], then, we opted to include this population in our work since our proposal was to observe a more ‘closer to reality’ cohort when designing the study. “*

**19. The limitations of this study need to include more details. The number of patients should be included in future studies as 10 patients may not be enough to draw firm conclusions. More females are needed in the future to support data comparisons. Similarly, the limitations should include the time of bite and antivenom doses.**

Response: We appreciate the insightful feedback regarding the limitations of our study, and we have taken steps to enhance the details provided in this regard. Firstly, we acknowledge the importance of sample size in drawing robust conclusions.

- While our study included 10 patients, recognizing the potential for limitations in generalizability, we have now explicitly addressed this concern in the limitations section. Future studies will undoubtedly benefit from larger sample sizes to strengthen the statistical power and enhance the reliability of our findings.
- Regarding gender representation, we concur with the reviewer's observation that a more balanced distribution is desirable for comprehensive data comparisons. We have incorporated this consideration into the limitations, emphasizing the need for increased female representation in future studies to support more nuanced and gender-specific analyses.
- The timing of snakebite and the administration of antivenom are crucial factors, and we appreciate the reviewer's keen focus on these aspects. In response, we have augmented the limitations section to explicitly include the time of snakebite, interpreted as the duration between the accident and hospital admission. However, we would like to clarify that the limitation related to antivenom doses was not included, as our study's design involved urine collection at a single time point before antivenom administration. This ensures that the treatment does not impact the presented analysis. Furthermore, we have reiterated in the limitations section that all patients received treatment at the same hospital, minimizing potential variations in treatment approaches that could influence complication outcomes.

We are grateful for the constructive feedback, and these refinements contribute to a more comprehensive acknowledgment of the study's limitations, guiding future research directions and interpretations.

See below the improvements regarding limitations (lines 586 -598):

*“Moreover, the naturally high variability in urine poses a challenge for analyzing these samples, particularly for biomarker research [19,20,51]. Various factors are already known to influence the urinary protein landscape, such as the patient’s sex and age, the timing of sample collection, the presence of comorbidities, dietary composition, the volume of venom inoculated, and even the time between the bite and hospital care [22,68,89]. Thus, the results of this work are also limited by the small size cohort of AKI patients, the few female patients observed, the presence of AKI patients with hypertension, and the analysis of only AKI at Stage 2; thus further studies with larger cohorts observing these aspects are needed to validate and reinforce the results presented in our study. These limitations cited impose still unknown effects on the data presented, especially the presence of hypertensive patients. Hypertension frequently causes renal damage, the extension of it it’s correlated to several factors, such as the individual susceptibility, the degree of hypertension, or even if it's being properly treated or not [91]. These possible injuries might lead to a higher abundance of some proteins and create a bias for these in the data. However, hypertension is a common comorbidity in the general population and among AKI patients [93], then, we opted to include this population in our work since our proposal was to observe a more ‘closer to reality’ cohort when designing the study.”*

**20. I suggest the authors avoid including small paragraphs in the discussion. It's better to combine all the information relating to each theme in a single paragraph.**

Response: Thank you for your suggestion. We have revised the discussion section by consolidating information related to each theme into single, cohesive paragraphs.

**21. Finally, I strongly recommend authors thoroughly proofread this article as there are some typographical and grammatical errors.**

Response: We have diligently conducted a thorough proofreading process to rectify any grammatical and typographical errors present in the article. We appreciate your attention to detail and the importance of linguistic precision in scientific writing. If you have specific instances or areas where errors persist, please feel free to point them out, and we will address them promptly. Your feedback is invaluable in ensuring the overall quality and clarity of our manuscript.
